# Procedure for Calibrating the Z-axis of a Confocal Microscope: Application for the Evaluation of Structured Surfaces

**DOI:** 10.3390/s19030527

**Published:** 2019-01-27

**Authors:** Chen Wang, Jesús Caja, Emilio Gómez, Piera Maresca

**Affiliations:** Escuela Técnica Superior de Ingeniería y Diseño Industrial, Universidad Politécnica de Madrid, Ronda de Valencia 3, 28012 Madrid, Spain; chen.wang@alumnos.upm.es (C.W.); jesus.caja@upm.es (J.C.); emilio.gomez@upm.es (E.G.)

**Keywords:** areal surface texture, confocal microscopy, uncertainty evaluation, structured surface

## Abstract

This work describes a method for the metrological characterization of structured surfaces using a confocal microscope. The proposed method is based on the calculation of texture parameters established in ISO 25178-2:2012. To ensure the traceability of these parameters, a procedure for the calibration of the Z-axis of the confocal microscope is proposed. The calculation of uncertainty associated with each parameter employs the Monte Carlo method, as well as the concept of a virtual instrument. The validity of the algorithms has been verified through the use of synthetic data provided by the National Institute of Standards and Technology (NIST) and physical standards, with minimum differences being obtained between the certified values and calculated or measured values. Finally, using the proposed method, the topography of a structured surface manufactured by laser machining is evaluated, obtaining the most used roughness parameters, as well as their measurement uncertainties and possible correlations. In general, it can be affirmed that it is possible to obtain metrologically reliable results with the proposed method.

## 1. Introduction

The study and design of structured surfaces represents a new frontier for science, because unlike surfaces produced by conventional methods, they have specific functional performances [1,2], such as hydrophobic or hydrophilic [3,4], anti-corrosive [5,6], anti-icing [7,8,9], and osseointegration [10,11] properties, to name a few. As indicated by Wong et al. and MacAulay et al. [12,13], it is essential to develop efficient, high precision surface characterization methods for structured surfaces.

Research in this field is gaining progressively more interest due to its growing importance in various scientific and industrial application areas; such as biomedical engineering, telecommunications industries, aerospace, as well as in the photovoltaic energy field [14]. In particular, the study of these phenomena on the micro- and nano-metric scale represents an advance in scientific research in different fields like electronics, tribology, optics, etc. [15]. Therefore, it is essential to be able to perform an accurate metrological characterization of these surfaces in the micro- and nano-metric range.

In the field of surface metrology, the ISO 25178 standard series of the Geometrical Product Specifications (GPS) system establishes the quantitative methods for the characterization of the roughness (or texture) of the surfaces, that is, the parameters and characteristic curves involved in the measurement, as well as the measuring instruments and reference standards used.

The studies made by authors such as De Chiffre and Lonardo [16,17,18] around the beginning of the 21st century, in addition to showing new trends in the metrological characterization of surfaces, show the need for new measurement procedures, instruments, and innovations for the measurement of roughness parameters, so that the new needs for tolerance, traceability, and calibration represent the beginning of a new phase of research in this field.

In the last 20 years, many scientific studies have been developed from these needs, and much progress has been made on the evaluation of areal surface texture parameters. One of the most recent studies, by Todhunter et al. [19], shows the results of an international survey on the use of roughness parameters in industry. The survey revealed that a great variety of parameters has been used in the industrial field in recent years, as indicated in the different ISO standards [20,21,22,23,24]. Compared with a similar survey conducted in 1999 by Professor De Chiffre [25], it is evident that not only the use of the profile parameters has increased, but the adoption of the new areal surface texture parameters is very significant, as indicated in ISO 25178-2, since its entry into force in 2012 [24].

With the introduction of areal surface texture parameters in the scientific and industrial field [26,27], commercial software packages have been developed in parallel with their calculation, such as those implemented by the National Physical Laboratory (NPL) [28], and the use of optical instruments for the implementation of these measures has increased [29,30,31,32], with confocal microscopes being one of the most used instruments.

In the scope of this work, a method was developed to traceably characterize the structured surface of a certain material from analysis of the surface roughness parameters obtained through the use of a Leica brand confocal microscope, model DCM 3D.

To ensure the traceability of the 3D measurements made with this instrument, it needs to be calibrated. In order to do so the accurate measurement of the Z-axis is fundamental for the quantitative analysis of the texture of the surfaces (height parameters). In the most recent scientific literature, different ways of calibrating the Z-axis of instruments with an optical axis, such as the confocal microscope, have been found, either by comparison with a reference instrument or by in situ methods.

Undoubtedly, one of the most common techniques for surface height calibration is using a flat reference area with one or more rectangular grooves. The use of this type of standard allows comparison of the measured value with the certified value of a step-height standard, which gives an indication of how well the instrument can make measurements of the difference in height of nominally flat areas. The use of step standards is common in many scientific works [33,34,35]. In particular, the work developed by Seppä et al. [36] employs a step standard to determine the scale factor associated with the Z-axis, as well as the linearity thereof, so that a behavior equation of the scale factor calculated through a polynomial of 3 degrees is determined. Additionally, the possible noise associated with the Z-axis readings due to vibrations, the effect of the light intensity, as well as the reflectivity of the sample are analyzed. The authors Udupa et al. [37] analyze the influence of the number of data captured, as well as the optical system, illumination, object, and mechanical system. Likewise, the inclination of the sample has been analyzed, with the best position being the lowest inclination (4–8°). Additionally, Nouira et al. [38] study what are the effects of the material, the roughness of the surface, the color, the inclination of the sample, the shape of the geometry in the results, and the speed of scanning, depending on the working range of the probe. It is determined that the greater the work field, the lower the influence of the previous parameters. In the works of Giusca et al. [39,40] it is determined that placing the measurement perpendicular to the optical axis is critical for the realization of the measurements. Therefore, different grooves are measured and a behavior curve is obtained, taking into account the repeatability of the measurements. In Reference [41] a method to calibrate the X, Y, and Z axes at the same time using a 2d square array of dots standard inclined at 30° with respect to the measuring table is described. Other studies, such as that by De Chiffre et al. [42], show the need to obtain traceability with 3D techniques at the micrometric and nanometric scale, by presenting a new device through which the calibration of the z-coordinate is allowed for different magnifications without needing to reposition the measurand, which allows traceability to be transferred to 3D techniques.

Finally, in the works of Corbett et al. and Korpelainen et al. [43,44], a laser interferometer is used for measuring the vertical displacement of the equipment head and comparing it with the equipment measurements.

One of the most important references in the field of scientific literature for the calibration of these instruments is the document “Calibration of the metrological characteristics of Imaging Confocal Microscopes (ICMs) Measurement Good Practice Guide No. 128” [45], where methods appear for the determination of the characteristic metrological parameters required for the measurement and calibration of confocal microscopes, in accordance with the international standards for the specification of areal surface texture [46].

Unlike the methods described above and presented by other authors [47,48], the procedure presented in this study to provide traceability for roughness parameters is based on the concept of virtual CMM (Coordinate Measuring Machine) [49], which uses the variability in the *z*-coordinates captured by the instrument, thus simulating the actual behavior of the equipment.

Following the indications in the ISO 25178 series standards, different calculation algorithms were developed in Matlab for the determination of areal surface texture parameters [24]. To test the validity of the developed algorithms, a roughness standard was measured, whose arithmetic mean height *Sa* was 1.003 µm. In addition, synthetic data provided by the National Institute of Standards and Technology (NIST) was used [50]. Finally, a structured surface produced at the “Centro Laser” of the Universidad Politécnica de Madrid was evaluated.

The proposed model provides a reliable and traceable way to superficially characterize any measurand via the *S* surface roughness parameters.

## 2. Measurement Method

The procedure used to characterize surface roughness parameters, from the coordinates (x, y, z) corresponding to an area to be evaluated, is structured in the following steps:

(a) Obtaining the surface evaluated by a confocal microscope. The file provided by the equipment contains the sampled x and y coordinates and the digitized z coordinates $\left(x_{r},\;y_{r},\;z_{r}\right)\;$.
(b) Cropping the surface evaluated. As the evaluated area is not square, this is cropped so an area of *l* × *l* will be obtained. The length *l* will be equal to the nesting index L (Section 2 item e)
(c) S Filter. The area evaluated is filtered using a low-pass filter with a nesting index *S*_1_. The primary area with coordinates $\left(x_{rS},\;y_{rS},\;z_{rS}\right)$ is obtained. The nesting index is an extension of the cut-off wavelength concept employed in the profile roughness measurements (2D). The *S*_1_ filter nesting index determines the maximum sampling distance and eliminates small-scale lateral components of the surface, for example measurement noise or small features. The value of the nesting index *S*_1_ is determined according to the recommendations of the ISO 25178-3:2012 [51], and a Gaussian filter according to ISO 16610-61:2015 [52] is used.
(d) Form removal. Since it is very laborious to place the measurand entirely perpendicular with respect to the confocal microscope optical axis, and also due to the form errors characteristic of the measurand, its nominal form needs to be removed by adjusting the surface to a basic geometrical feature (e.g., a plane or sphere). When the deviation of the adjusted component is very small, it can be corrected by subtracting the adjusted component from the measured coordinates. However, in other situations the adjusted component has to be rotated so that its normal coincides with the instrument optical axis. After removing the shape, the S–F surface with coordinates $\left(x_{rSF},\;y_{rSF},\;z_{rSF}\right)$ is obtained. When the element to be adjusted is a plane, it can be represented mathematically as:
  (1) ax + by + cz + d = 0.
  The coefficients a, b, c, d can be calculated using the least squares or minimum zone method. The plane that best fits the coordinates $\left(x_{i},\;y_{i},\;z_{i}\right)$ is:
  (2) $$ax_{i}\;+\;by_{i}\;+\;cz_{i}\;+\;d\;+\;e_{i}\;\approx \;0$$
  where $e_{i}$ are the residuals of the adjustment. If the least square problem is solved, a method that solves homogeneous equations must be used [53]. Using the same method, it is possible to remove the form error of the evaluated surface by adjusting it to other basic geometrical features (e.g., spheres, cylinders, cones, or quadrics).
(e) L Filter. The *S*–*F* Surface is filtered using a high-pass filter with a nesting index *L* to give the *S*–*L* surface with coordinates $\left(x_{rSFL},\;y_{rSFL},\;z_{rSFL}\right)$. The nesting index *L* determines the size of the primary surface and eliminate large-scale lateral components of the surface. The value of the nesting index *L* is determined according to ISO 25178-3:2012 [51]. A Gaussian filter is used according to the ISO 16610-61:2015 standard [52].
(f) Obtaining the areal parameters, according to the ISO 25178-2:2012 standard [24]. For the determination of the height parameters *Sa*, *Sq*, *Ssk*, *Sku*, an analytical calculation method (Simpson’s rule) is used, instead of using the discrete calculation formulas [54] (Equations (6)–(9)), so that it is possible to improve the accuracy of the calculations.
  (3) $$Sp\;=\underset{1\leq i\leq n_{x}n_{y}}{\max}z_{pi}\;\Rightarrow \;\mathrm{calculated}\;\mathrm{in}\;\mathrm{the}\;\mathrm{evaluated}\;\mathrm{area}$$
  (4) $$Sv\;=\;\underset{1\leq i\leq n_{x}n_{y}}{\max}z_{vi}\;\Rightarrow \;\mathrm{calculated}\;\mathrm{in}\;\mathrm{the}\;\mathrm{evaluated}\;\mathrm{area}$$
  (5) Sz = Sp + Sv ⇒ calculated in the evaluated area
  (6) $$Sa\;=\;\frac{1}{A}\iint_{A}\left|z\left(x,y\right)\right|d_{x}d_{y}\;\approx \;Sa\;=\;\frac{1}{n_{x}\cdot n_{y}}\sum_{j=1}^{n_{y}}\sum_{i=1}^{n_{x}}\left|z\left(i,j\right)\right|$$
  (7) $$Sq\;=\;\sqrt{\frac{1}{A}\iint_{A}z^{2}\left(x,y\right)d_{x}d_{y}\;}\;\approx \;Sq\;=\;\sqrt{\frac{1}{n_{x}\cdot n_{y}}\sum_{j=1}^{n_{y}}\sum_{i=1}^{n_{x}}z^{2}\left(i,j\right)}$$
  (8) $$Ssk\;=\;\frac{1}{Sq^{3}}\frac{1}{A}\iint_{A}z^{3}\left(x,\;y\right)d_{x}d_{y}\;\approx \;Ssk\;=\;\frac{1}{Sq^{3}}\frac{1}{n_{x}\cdot n_{y}}\sum_{j=1}^{n_{y}}\sum_{i=1}^{n_{x}}z^{3}\left(i,\;j\right)$$
  (9) $$Sku\;=\;\frac{1}{Sq^{4}}\frac{1}{A}\iint_{A}z^{4}\left(x,\;y\right)d_{x}d_{y}\;\approx \;Sku\;=\;\frac{1}{Sq^{4}}\frac{1}{n_{x}\cdot n_{y}}\sum_{j\;=\;1}^{n_{y}}\sum_{i\;=\;1}^{n_{x}}z^{4}\left(i,\;j\right)$$

The parameters *Sp*, *Sv*, and *Sz* evaluate the amplitude of the measured area (peak and valley distances), *Sa* is used to calculate the arithmetic mean height, *Sq* evaluates the variance of the amplitude distribution function (ADF) of the area, *Ssk* determines the shape of the distribution of the surface height evaluated, and *Sku* evaluates the sharpness of the distribution of the evaluated surface height.

These parameters were selected for being the most representative and of greatest interest in the 3D characterization of surfaces according to Todhunter et al. [19], where the most used parameter turns out to be *Sa*, followed by the parameters *Sq* and *Sz*.

## 3. Validation of the Algorithms

Before developing the uncertainty calculation model, the accuracy and validity of the algorithms implemented was determined. For this purpose, reference data sets [55] were used, which represent the coordinates (x, y, z) of a reference surface. The databases provided by NIST on their Internet based Surface Metrology Algorithm Testing System were used [50]. The reference coordinates are governed following the indications of ISO 25178-71:2017 [56] according to reference data Type S1.

**Figure 1:**
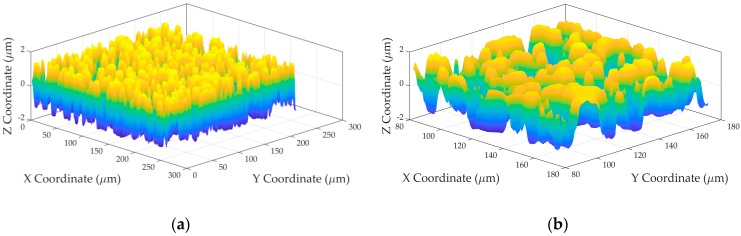
SG_3-3 reference surface (**a**) Unfiltered; (**b**) Filtered with *c* = 0.08 mm.

Different tests were done with the reference data provided by NIST, with Figure 1 showing one of the reference surfaces (SG_3-3), unfiltered, and after treatment with a high-pass filter with a nesting index *L* of 0.08 mm. Table 1 shows the results of this test. The difference (*Q*_1_) in absolute values between the NIST reference values and the values provided by the developed algorithms was evaluated.

(10) $$Q_{1}\;=\;\left|Sxx_{\mathrm{reference}}\;-\;Sxx_{\mathrm{calculated}}\right|$$

The results show the maximum percentage difference was 0.0232%, so it can be indicated that the algorithms for calculating developed roughness parameters and surface filtering behaved satisfactorily.

## 4. Roughness Parameters Calculation Model: Assurance of Traceability

### 4.1. Mathematical Model

To ensure the traceability of the roughness values determined by the confocal microscope, a calibration procedure for the Z-axis is needed, so that the unit of length can be disseminated.

There are different metrological models for this calibration [35,57]. This study used the following mathematical model to correct for the roughness parameters:

(11) $${\mathrm{Sparameter}}_{c}=C\cdot \left({\mathrm{Sparameter}}_{m}+\delta {\mathrm{Sparameter}}_{\mathrm{noise}}+\delta {\mathrm{Sparameter}}_{\mathrm{light}}+\delta {\mathrm{Sparameter}}_{\mathrm{tilt}}\right)$$

where ${\mathrm{Sparameter}}_{c}$ represents the corrected roughness parameter, C is the Z-axis calibration coefficient, ${\mathrm{Sparameter}}_{m}$ is the measured roughness parameter taking into account the measurement repeatability, $\delta {\mathrm{Sparameter}}_{\mathrm{noise}}$ takes the noise in the measurements into account (from the instrument and environment) and $\delta {\mathrm{Sparameter}}_{\mathrm{noise}}$ and $\delta {\mathrm{Sparameter}}_{\mathrm{tilt}}$ take the measurement reproducibility into account after changing the measurement conditions (different sample illumination and tilt, respectively).

The methods used to obtain the values in Equation (11) are described below. These values were used to perform the different measurement experiments with the confocal microscope, which are presented in Section 5.

**Figure 2:**
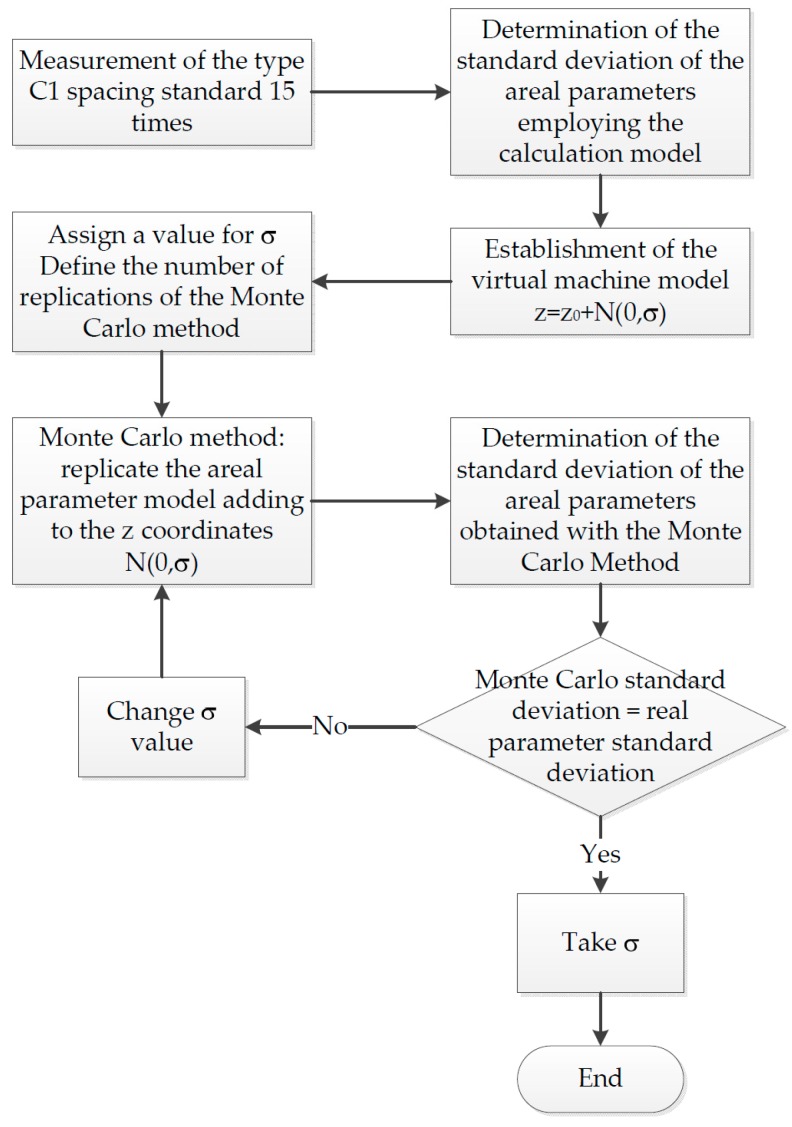
Flow chart for the determination of σ.

**Figure 3:**
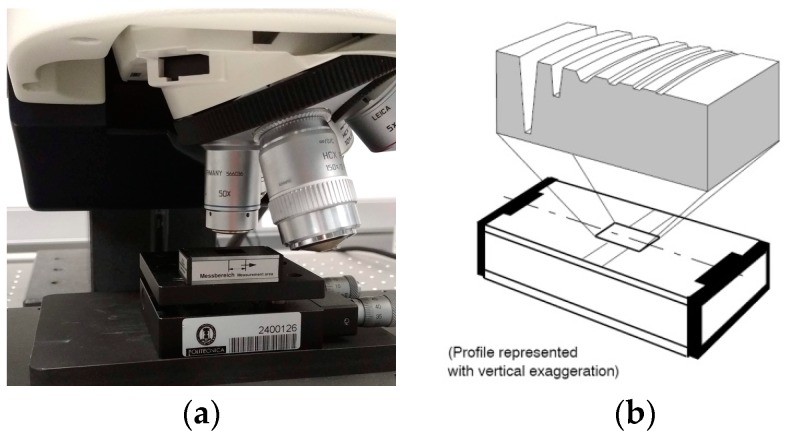
Step height standard (**a**) Experimental setup; (**b**) Groove geometry, source: HALLE Präzisions-Kalibriernormale GmbH [58].

(a) Measurement repeatability: For its analysis, an experiment was conducted in which a roughness standard was measured 15 times, in this case, a type C1 spacing standard, grooves with a sine wave profile, with a measurement area of 0.8 × 0.8 mm. The surface was first filtered employing a low pass filter with an 8 µm nesting index, then later with a high pass filter with a nesting index of 800 µm. The lighting and the tilt of the measuring table were kept constant during the measurements. The values of the parameters *Sa*, *Sq*, *Ssk*, *Sku*, *Sz*, *Sp*, and *Sv* were determined in each of the measurements and the standard deviation of the 15 measurements obtained. These experimental values are shown in Table 2.
  Bearing in mind that measurements with confocal equipment takes a long time (20–35 min for a measurement area of 0.8 × 0.8 mm), the technique used in the virtual CMM was adopted [49], so the real behavior of the equipment was simulated. Using this technique, an uncertainty contribution was added to the captured coordinates: $z\;\approx \;z_{0}\;+\;\delta_{z}$. The variability due to $\delta_{z}$ was estimated by observing the values provided by the equipment. The study assumed that the variability associated with the confocal microscope *z*-coordinates used was characterized by a normal of mean zero and standard deviation σ, that is $z\;\approx \;z_{0}\;+\;N\left(0,\sigma^{2}\right)$, that this variability was the same for all the coordinates, and that there was no correlation between evaluated coordinates.
  Figure 2 shows the flowchart of how to determine the dispersion σ to be used in the *z*-coordinates. A Monte Carlo simulation model was implemented, which used the algorithms developed in Section 2, and simulated this model with a number of replications *N* = 1000. In each trial, the standard deviation value was varied, until variability values similar to those in Table 2 were obtained, for each of the roughness parameters. After the different trials, it was verified that the standard deviation that best adapted to the results was 0.1 µm.
(b) Z-axis calibration coefficient (C): This was obtained using a step height standard with trapezoidal grooves (Figure 3a,b). Four grooves were measured, those of nominal values 24, 7, 2, and 0.7 µm as they were the measurement range of the measurands analyzed in this work. The certified values (provided by an accredited laboratory) are shown in Table 3.
  A procedure to evaluate the previous step standard heights was used. This procedure is based on initially adjusting the upper surface of the step height standard to a reference plane and then rotating it. Next, the mean distance was determined between this reference plane and the lower planes of the different grooves [59]. Each groove was measured using a Leica confocal microscope, model DCM-3D, with a 50× objective. The standard was measured 10 times. Table 4 shows the mean results obtained, the difference between the measured and certified value, the correction coefficient value (the quotient of the certified value and measured value) and the correction coefficient standard uncertainty, by using the standard uncertainty and measurement repeatability of the step heights as sources of uncertainty.
  It was observed that for the nominal values of 2 and 0.7 µm, the differences with the certified value were in the order of nanometers. Usually, a global correction coefficient is used C, with a value equal to 1 for the whole scale. The difference in values obtained with respect to the global value was included in the standard uncertainty of this coefficient. From the values of the correction coefficients (Table 4, Line 6) and their standard uncertainty (Table 6, Line 7), a global correction coefficient equal to 1 was obtained, which responded to a uniform distribution of limits 1 ± 0.03 [−].
(c) Measurement noise ($\delta {\mathrm{Sparameter}}_{\mathrm{noise}}$). An experiment was carried out measuring a flatness standard (metallic coated glass) multiple times (n = 15) with flatness λ10, and the roughness parameter *Sq* was analyzed both with and without averaging the coordinates. To determine the standard uncertainty associated with the noise from each parameter, the indications of the document “Calibration of the metrological characteristics of Imaging Confocal Microscopes (ICMs) Measurement Good Practice Guide No. 128” [45] were used. The standard uncertainty associated with the noise of this parameter was calculated as:
  (12) $$u\left(\delta {\mathrm{Sparameter}}_{\mathrm{noise}}\right)\;=\;\sqrt{\frac{Sq_{\mathrm{un}\text{-}\mathrm{averaged}}^{2}-Sq_{\mathrm{averaged}}^{2}}{1-\frac{1}{n}}}.$$
  This variable had a normal distribution, with a most probable value equal to zero and standard uncertainty equal to the value provided by the previous equation. Table 5 shows the results obtained in the experiment:
(d) Light intensity variation ($\delta {\mathrm{Sparameter}}_{\mathrm{light}}$). The determination of the contribution made by this was by measuring in reproducibility conditions with a type C1 spacing standard, with grooves and a sine wave profile. The standard was measured 10 times, with the lighting value provided by the equipment changed each time. The light intensity range should be selected so that the sensor of the microscope will not be saturated and always receives signal. Over the 10 measurements, the nominal illumination value was changed by ±15% The standard deviation of the roughness parameters was determined from the 10 measurements (Table 6). This contribution was considered because it is not possible to know with absolute certainty which is the optimum level of illumination.
(e) Variation of sample inclination ($\delta {\mathrm{Sparameter}}_{\mathrm{tilt}}$). The determination of the contribution made by this was by measuring in reproducibility conditions with a type C1 spacing standard, with grooves and a sine wave profile. The standard was measured 10 times, and the inclination of the tilt table changed in each (over the 10 measurements, the table inclination changed ±1° with respect to the horizontal). The standard deviation of the roughness parameters was determined from the 10 measurements (Table 6). This contribution was considered because it is practically impossible to place the measurand perpendicular to the equipment optical axis.
  For the previous contributions ($\delta {\mathrm{Sparameter}}_{\mathrm{light}}$ and $\delta {\mathrm{Sparameter}}_{\mathrm{tilt}}$) it was assumed these would have a normal distribution with a mean of zero and standard deviation as shown in Table 6). The standard uncertainties were calculated as type A uncertainties according to the following expression:
  (13) $$u\left(\delta {\mathrm{Sparameter}}_{\mathrm{light}}\right)\;=\;\sqrt{\frac{s_{{\mathrm{Sparameter}}_{\mathrm{light}}}}{n}},\;\;u\left(\delta {\mathrm{Sparameter}}_{\mathrm{tilt}}\right)\;=\;\sqrt{\frac{s_{{\mathrm{Sparameter}}_{\mathrm{tilt}}}}{n}}$$
  where S is the standard deviation of the analyzed parameters and n represent the numbers of times the roughness was measured (typically 1).

### 4.2. Model for Calculating Uncertainty

To calculate the uncertainty associated with the corrected roughness parameters (Equation (11)), the Monte Carlo method was used. This is widely used as a numerical resolution method in various metrology fields [60,61,62,63].

The recommendations used were those established in the Supplement 1 to the “Guide to the expression of uncertainty in measurement”—Propagation of distributions using a Monte Carlo method [64] and Supplement 2 to the “Guide to the expression of uncertainty in measurement”—Extension to any number of output quantities [65] for the development of algorithms for calculating uncertainty.

The algorithms for calculating the uncertainty associated with the corrected roughness parameters are detailed in the following steps.

(a) Definition of the output quantities. Corrected roughness parameters ${\mathrm{Sparameter}}_{c}$.
(b) Definition of input quantities. The Z-axis calibration coefficient (C) sampled z-coordinates of the surface and the $\delta {\mathrm{Sparameter}}_{\mathrm{noise}}$, $\delta {\mathrm{Sparameter}}_{\mathrm{light}}$, and $\delta {\mathrm{Sparameter}}_{\mathrm{tilt}}$ parameters. Sources of uncertainty that considered the variability of the parameters and coordinates measured by the confocal microscope were examined, as well as a factor for traceability for measurements on the Z-axis.
(c) Assignment of the probability density functions (PDF) to the input variables. For the input variables in the previous section, the following was established:
  The coefficient C had a uniform distribution of limits 1 ± 0.03, calculated in Section 4.1 item b.
  The variability associated with the *z*-coordinates was according to a normal distribution, whose mean was the coordinate captured by the equipment and with a standard uncertainty of 0.1µm, calculated in Section 4.1 item a.
  The measurement noise had a normal distribution of zero mean and standard uncertainty equal to 0.0090 µm, calculated in Section 4.2 item c.
  The light intensity variation had a normal distribution of zero mean and standard deviation (depending on the parameter) as shown in Table 6.
  The table tilt variation had a normal distribution of zero mean and standard deviation (depending on the parameter) as shown in Table 6.
(d) Propagation. The Supplement 1 to the GUM (Guide to the expression of uncertainty in measurement), Section 7.2.3 [65], suggests a reduced number of trials (M) can be used for complex models. Based on this recommendation, the mean values and their standard uncertainty after replicating the model *M* trials could be taken as u(y) and y respectively, and be assigned to Gaussian PDF $g_{Y}\left(\eta \right)\;=\;N\left(y,\;u^{2}\left(y\right)\right)$. A total of 5000 trials were conducted to obtain the experimental results, requiring 2–3 h of calculation in a computer with an Intel(R) Core(TM)-i7-6700HQ and memory of 16 GB.
(e) Results. From the *M* trials of the models, the most probable values and standard uncertainties of the roughness parameters were determined. To determine the interval of uncertainty, the minimum interval method was used [66,67]. Finally, when the calculation models provided multiple results, the covariance matrix was determined, as well as the matrix of correlation coefficients according to that established in Supplement 2 of the GUM [65].

## 5. Experimental Verification

### 5.1. Checking the Type C1 Spacing Standard

Before measuring the structured surface, the type C1 spacing standard was evaluated with a certified *Sa* value of 1.003 µm and an expanded uncertainty (coverage factor k = 2) of 3% of this value.

As indicated, the equipment used for the measurement was a Leica confocal microscope, model DCM-3D, which uses an LED illumination source, with a wavelength of 460 nm of episcopacy type. The measurements were made with the confocal mode of the equipment. The objective used was an infinity corrected 50×, with a NA (Numerical Aperture) of 0.5, and a working distance of 8.2 mm. This objective gave good amplification with a high point acquisition speed. The field of measurement of this objective was 0.25664 mm × 0.19090 mm, with a lateral sampling distance 0.332 m and a vertical resolution of less than 3 nm. The total measurement area of this equipment was 114 mm × 75 mm.

The laboratory temperature where the measurements were made was kept at 20 ± 1 °C.

The measured area was approximately 1 mm × 1 mm, with a stitching of 5 (horizontal) per 7 (vertical) measuring fields being necessary to be performed. The area evaluated for the surface roughness was reduced to 0.8 mm × 0.8 mm.

The evaluation area was selected as indicated in the ISO 4288 standard [68], which recommends a cut-off of 0.8 mm for a roughness *Ra* of 1.003 (for this type of standard, the values of *Ra* and *Sa* are similar). In accordance with the standard ISO 25178-3 [51], the S-filter nesting index value was established at 0.008 mm (used when the sampling distance was ≤2.5 µm) and the value of the L-filter nesting index was 0.8 mm (bandwidth ratio between the F-operation or L-filter and S-filter nesting indices of 300). As there was a significant probability of obtaining outliers in the sample, a high S nesting index value was used (considering the sampling distance, an S-filter nesting index of 0.0025 was used) to eliminate these outliers. Outliers appear when the equipment light intensity is very high, and the CCD sensor becomes saturated, which alters the peak position, giving incorrect values for the z-coordinate.

**Figure 4:**
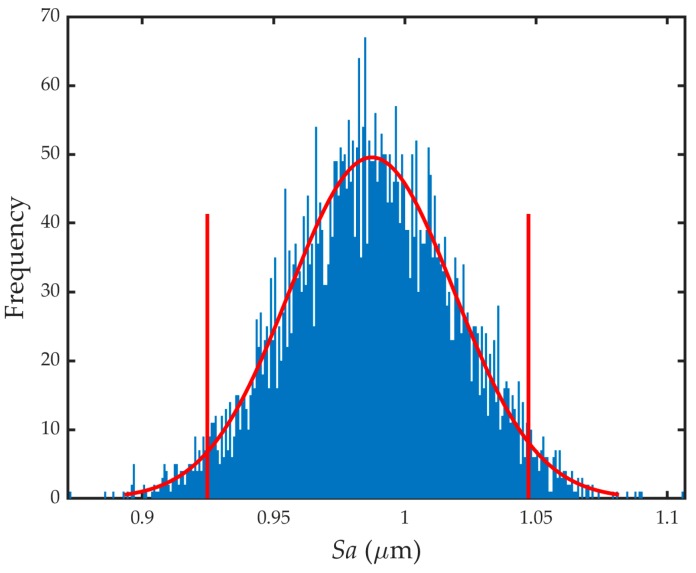
*Sa* histogram.

Table 7 shows the results obtained (most probable value, standard uncertainty, and 95% coverage interval), as well as the certified *Sa* parameter values. For the calculation of uncertainty, 5000 trials of the model were conducted. As can be seen, the difference between the certified and measured value was 1 nm, which in percentage terms was 0.1%. The standard uncertainty calculated had a value of 32 nm with a normal distribution, as shown in Figure 4.

The results obtained confirm that the surface roughness parameter calculation model and calculation of uncertainties was correct and capable of ensuring the traceability of the measurements.

### 5.2. Structured Surface

**Figure 5:**
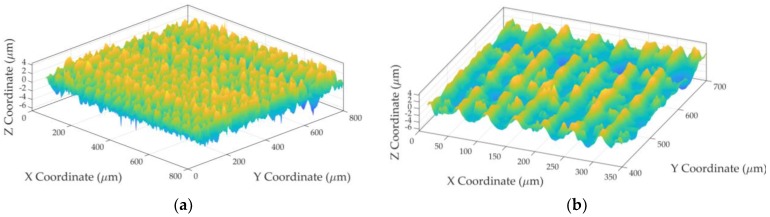
Structured surface parameters (**a**) Evaluated surface; (**b**) Detail.

A structured surface like the one shown in Figure 5 was evaluated below. This titanium surface was produced at the “Centro Laser” of the Universidad Politécnica de Madrid. It was manufactured with a laser and given grooves in the horizontal and vertical directions.

The Leica confocal microscope, model DCM-3D described in the previous section, was used and the environmental temperature maintained at 20 ± 1 °C for the measurements.

The roughness in the main direction was initially evaluated, and an *R_a_* of 1.2 µm obtained, so a cut-off of 0.8 mm was used again. The measured area was 1 mm × 1 mm approximately, with the field stitching measuring 5 (horizontal) per 7 (vertical). The area evaluated for the surface roughness was reduced to 0.8 mm × 0.8 mm. An S-filter nesting index of 0.008 mm and an L-filter nesting index of 0.8 mm were used, for the same reasons as indicated in Section 5.1.

**Figure 6:**
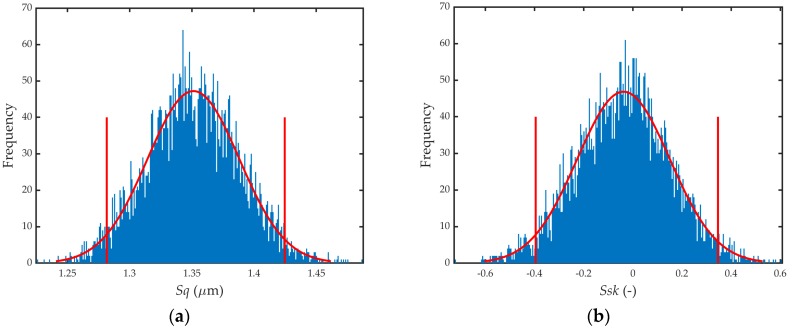
Structured surface parameters (**a**) *Sq* histogram; (**b**) *Ssk* histogram.

Table 8 shows the results obtained (most probable value, standard uncertainty, and 95% coverage interval). A total of 5000 trials were used to calculate the uncertainty by the Monte Carlo method. As can be seen, the standard uncertainty of the parameters was at least an order of magnitude smaller than the parameter itself (except for *Ssk*). Figure 6a,b show the histograms of the parameters *Sq* and *Sku*, which are reasonably similar to a normal distribution. The same behavior was seen for the rest of the parameters.

Table 9 shows the correlation coefficients between the roughness parameters. It can be seen that all the parameters are correlated with low correlation parameters.

## 6. Conclusions

This work was to develop a method for characterizing surface topographies traceably using a confocal microscope. The method included the following steps: pre-processing of data, assurance of the traceability of the measurements, determination of roughness parameters, verification of proprietary development calculation algorithms, and evaluation of contributions to uncertainty. The concept of a virtual machine was used to calculate the uncertainty associated with the z-coordinates and the characteristic 3D roughness parameters. The effectiveness of the proposed methods was verified via two methods: physical measurement of the roughness standards and verification with synthetic data provided by the NIST. The difference between the certified and measured values for the most characteristic parameter *Sa* was of the order of nanometers. The differences between the values calculated using the synthetic data with respect to the certified values were practically zero for all the parameters. The characterization of a structured surface manufactured by laser machining provided practical verification for the applicability of the proposed method.

## Figures and Tables

**Table 1:** Reference values vs calculated values (National Institute of Standards and Technology (NIST) SG_3-3 unfiltered and filtered surface).

	Unfiltered Surface				Filtered Surface			
Parameter	Reference Value	Calculated Value	*Q*_1_ (× 10^−6^)	Percentage Difference (%)	Reference Value	Calculated Value	*Q*_1_ (× 10^−6^)	Percentage Difference (%)
*Sa* [µm]	0.89928	0.899	0.5	0.0005	0.85828	0.858	0.2	0.0002
*Sq* [µm]	1.0	1.000	0.3	0.0003	0.96137	0.961	0.5	0.0005
*Ssk* [−]	0.01957	0.0196	0.5	0.0232	−0.0099	−0.0099	0.0	0.0001
*Sku* [−]	1.51103	1.5110	0.5	0.0003	1.56465	1.5647	0.3	0.0002
*Sz* [µm]	2.91342	2.913	0.0	3.5·10^−6^	3.23026	3.230	0.3	9.1·10^−5^
*Sp* [µm]	1.46487	1.465	0.1	8.7·10^−5^	1.5812	1.581	0.3	0.0002
*Sv* [µm]	1.44855	1.449	0.1	8.1·10^−6^	1.64906	1.649	0.0	2.1·10^−5^

**Table 2:** Standard deviation of areal parameters.

*Sa* [µm]	*Sq* [µm]	*Ssk* [−]	*Sku* [−]	*Sz* [µm]	*Sp* [µm]	*Sv* [µm]
0.006	0.007	0.018	0.009	0.071	0.071	0.050

**Table 3:** Certified values of the step height standard.

	Groove 1	Groove 2	Groove 3	Groove 4	Groove 5	Groove 6
Nominal value [µm]	75.00	24	7	2	0.7	0.2
Certified value [µm]	75.43	24.050	7.510	2.386	0.728	0.234
Expanded uncertainty (*k* = 2) [µm]	0.28	0.089	0.029	0.014	0.012	0.011

**Table 4:** Measurement values of the step height standard.

	Groove 2	Groove 3	Groove 4	Groove 5
Certified value [µm]	24.05	7.510	2.386	0.728
Measured value with CM [µm]	24.064	7.546	2.391	0.728
Standard deviation [µm]	0.016	0.054	0.028	0.019
Difference [µm]	−0.014	−0.036	−0.004	0.0001
Correction coefficient [−]	0.9994	0.9952	0.9980	1.0002
Standard uncertainty of the correction coefficient [−]	0.0006	0.0031	0.0049	0.0121

**Table 5:** Results obtained in the noise experiment.

	*Sq* [µm]
Mean without averaging the coordinates	0.0259
Mean with averaging the coordinates	0.0244
Uncertainty	0.009

**Table 6:** Experimental results from varying the light intensity and table inclination.

	*Sa* [µm]	*Sq* [µm]	*Ssk* [−]	*Sku* [−]	*Sz* [µm]	*Sp* [µm]	*Sv* [µm]
$s_{{\mathrm{Sparameter}}_{\mathrm{light}}}$	0.007	0.007	0.004	0.003	0.172	0.172	0.024
$s_{{\mathrm{Sparameter}}_{\mathrm{tilt}}}$	0.024	0.026	0.189	0.036	0.332	0.295	0.142

**Table 7:** Results of the measurement of the type C1 spacing standard.

	Parameter Estimation *y*	Standard Uncertainty *u*(*y*)	Shortest 95% Coverage Interval	
			Lower Limit	Upper Limit
*Sa*_certified_ [µm]	1.003	0.015	0.973	1.033
*Sa*_measured_ [µm]	1.002	0.032	0.942	1.065

**Table 8:** Results of the measurement of the structured surface.

	Parameter Estimation *y*	Standard Uncertainty *u*(*y*)	Shortest 95% Coverage Interval	
			Lower Limit	Upper Limit
*Sa* [µm]	1.106	0.033	1.043	1.170
*Sq* [µm]	1.351	0.037	1.282	1.425
*Ssk* [−]	−0.04	0.19	−0.40	0.35
*Sku* [−]	2.588	0.058	2.484	2.699
*Sz* [µm]	10.43	0.41	9.64	11.25
*Sp* [µm]	4.02	0.35	3.33	4.67
*Sv* [µm]	6.41	0.18	6.07	6.77

**Table 9:** Correlation coefficients.

	*Sa* [µm]	*Sq* [µm]	*Ssk* [−]	*Sku* [−]	*Sz* [µm]	*Sp* [µm]	*Sv* [µm]
*Sa* [µm]	1	0.381	0.012	0.449	0.257	0.124	0.348
*Sq* [µm]	0.381	1	0.003	0.480	0.274	0.140	0.378
*Ssk* [−]	0.012	0.003	1	0.005	0.020	0.010	0.004
*Sku* [−]	0.449	0.480	0.005	1	0.332	0.146	0.470
*Sz* [µm]	0.257	0.274	0.020	0.332	1	0.104	0.284
*Sp* [µm]	0.124	0.140	0.010	0.146	0.104	1	0.126
*Sv* [µm]	0.348	0.378	0.004	0.470	0.284	0.126	1
